# Intraoperative Posterior Chamber Irrigation to Enhance Vitreous Cavity Support during Phacoemulsification Cataract Surgery after Vitrectomy

**DOI:** 10.1155/2020/4132456

**Published:** 2020-02-17

**Authors:** Jiao Lyu, Peiquan Zhao

**Affiliations:** Department of Ophthalmology, Xinhua Hospital, School of Medicine, Shanghai Jiao Tong University, Shanghai, China

## Abstract

**Purpose:**

To report the effectiveness of an intraoperative posterior chamber irrigation technique to enhance vitreous cavity support during postvitrectomy phacoemulsification cataract surgery.

**Methods:**

The irrigation technique was performed during phacoemulsification cataract surgery on 10 postvitrectomy eyes (9 patients) with moderate or hard density cataracts and with low vitreous cavity support. A cohesive viscoelastic tamponade was applied to pressurize the anterior chamber to start the procedure. The vitreous cavity was then irrigated using a 26-gauge flushing cannula injecting balanced salt solution under the iris through the zonules, until the vitreous cavity pressure balanced and exceeded the anterior chamber pressure and viscoelastic flowed out from the corneal incision. Intraoperative performance with the irrigation technique, postoperative visual acuity, and anatomy, and complications were retrospectively evaluated.

**Results:**

The irrigation procedure instantly enhanced posterior segment pressure before capsulorhexis in 4 eyes, before phacoemulsification in 4 eyes, after phacoemulsification in 2 eyes, before intraocular lens implantation in 6 eyes, and after implantation in 3 eyes. Phacoemulsification cataract surgery was facilitated by the irrigation technique, with a stabilized anterior chamber and robust vitreous cavity support. No complications occurred intraoperatively and postoperatively. At a 3-month follow-up, favorable visual and anatomic outcomes were achieved in all eyes.

**Conclusions:**

The irrigation technique balanced the pressure of anterior and posterior segments. Thus, vitreous cavity support and anterior chamber depth were well stabilized during phacoemulsification cataract surgery in postvitrectomy eyes.

## 1. Introduction

Cataract is the most common complication of pars plana vitrectomy (PPV) used extensively to treat various posterior segment pathologies [1, 2]. Phacoemulsification cataract surgery is associated with more complications in an eye after PPV compared with nonvitrectomized eyes [3–6]. The prevalence of posterior capsule rupture (PCR) in nonvitrectomized eyes is 1.92 to 3.5% [7]. In contrast, the reported rates of PCR in vitrectomized eyes were up to 13.3%, accompanied with a higher rate of zonular dialysis, dropped nuclear fragments, and choroidal exudation [3, 4, 6, 8, 9]. The situation is relevant to anatomical changes of a vitrectomized eye that shows reduced vitreous gel support, possibly with defective zonular apparatus, and an impaired or fibrotic lens capsule [3–6]. Insufficient vitreous volume and fluid outflow from posterior segment to anterior chamber are associated with hypotony of the posterior segment during steps of the surgery when the anterior chamber is not irrigated. Insufficient vitreous cavity support also contributes to the disparity between the anterior and posterior pressures during fluid filling by phacoemulsification and aspiration (I/A), inducing an excessively fluctuating lens-iris diaphragm [3, 10–12]. These conditions may deteriorate preexisting zonular or capsular deficiency in vitrectomized eyes and trigger surgical complications [3, 4, 6, 10, 13]. Thus, fluid management between anterior and posterior segments is essential for maintaining vitreous cavity support to ensure positive cataract surgeries after PPV.

Current anterior segment strategies for a safer cataract surgery in postvitrectomy eyes involve accurate incisions, improved fluidics, and utilization of ophthalmic viscosurgical devices (viscoelastic) [14, 15]. These techniques help to stabilize the anterior chamber but do not restore intraoperative hypotony of the posterior segment. A technique of lifting the iris from the anterior lens capsule during phacoemulsification or I/A allows anterior irrigation to instantly reach the posterior segment through the zonules [16, 17]. However, the filling technique is restricted to phacoemulsification or I/A steps and it cannot be used in other surgical steps where irrigation is not performed. Complications associated with low vitreous support may occur not only during phacoemulsification or I/A but also after incisions, after capsulorhexis, and before or after intraocular lens (IOL) insertion [12, 13, 16], possibly requiring a pars plana infusion to support the vitreous cavity.

Herein, we described an intraoperative posterior chamber irrigation technique in which the anterior chamber was pressurized with viscoelastic and the vitreous cavity was irrigated across the zonules with balanced salt solution. We evaluated the effectiveness of the technique to restore vitreous cavity volume throughout phacoemulsification cataract surgery after PPV.

## 2. Materials and Methods

The posterior chamber irrigation technique (Supplementary [Supplementary-material supplementary-material-1]) was performed in ten eyes (nine patients) with postvitrectomy cataract between 2017 August and 2018 August, in Xinhua Hospital, Shanghai Jiao Tong University School of Medicine, Shanghai, China. All procedures were in accordance with the Declaration of Helsinki and approved by the Ethics Committee of Xinhua Hospital. Written informed consent was obtained from all patients.

Preoperative dilatation of the pupil was achieved using 0.5% tropicamide and 0.5% phenylephrine combination (Santen Pharmaceutical Co., Ltd., Osaka, Japan). Phacoemulsification surgeries were performed under peribulbar anesthesia. Briefly, routine surgical steps were as follows: A 3.0 mm clear corneal incision and a 1.0 mm side port were made. A 5.0 to 5.5 mm continuous curvilinear capsulorhexis was performed after cohesive viscoelastic (Medical Sodium Hyaluronate Gel, 17 mg/ml, Bausch and Lomb, Shandong Furuida Co., Ltd., China) was injected into the anterior chamber. Phacoemulsification using a “stop-and-chop” technique was performed with a 30-degree phaco tip (Constellation Vision System, Alcon Laboratories, Inc., Fort Worth, TX) in a continuous mode and torsional phaco power between 40 and 60% combined with a longitudinal phaco power between 0 and 30% depending on the density of the nucleus. Irrigation bottle height was between 65 and 80 cm H_2_O. A vacuum at 400 mm Hg and an aspiration flow rate between 35 and 40 cc/min were used. I/A for cortical clean-up was done with the same parameters. A single-piece or a 3-piece foldable acrylic IOL was implanted in the capsular bag. Viscoelastic was removed by I/A and incisions were hydrated.

**Figure 1:**
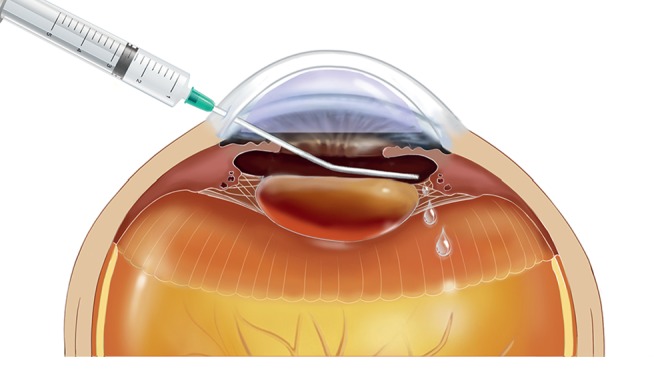
Schematic showing the intraoperative posterior chamber irrigation technique to restore vitreous cavity support during cataract surgery on vitrectomized eyes. With cohesive ophthalmic viscosurgical devices tamponade in the anterior chamber, a 26-gauge flushing cannula attached to a 5 ml syringe was directed under the iris, near the zonules and the lens equator, to maximally inject the balanced salt solution fluid across the zonular fibers, into the posterior chamber and vitreous cavity.

The posterior chamber irrigation procedure was applied before some major steps of cataract surgery when insufficient vitreous support was noted. Indications of insufficient vitreous support included soft scleral wall, excessive deep anterior chamber, and prominent posterior retropulsion of the iris-lens diaphragm after anterior chamber being inflated with viscoelastic or balanced salt solution [5, 13, 16]. The irrigation procedure started after the anterior chamber was inflated and pressurized by the cohesive viscoelastic. A 26-gauge flushing cannula connected to a 5 ml syringe filled with balanced salt solution was positioned into the anterior chamber through a 3.0 mm corneal incision (Figure 1). The flushing cannula tip was then directed under the iris near the zonules and the lens equator. Fluid was gently injected across the interspace of zonular fibers into the posterior chamber and then the vitreous cavity. Irrigation was terminated when the vitreous cavity pressure surpassed the anterior chamber pressure and viscoelastic flowed out of the corneal incisions.

**Figure 2:**
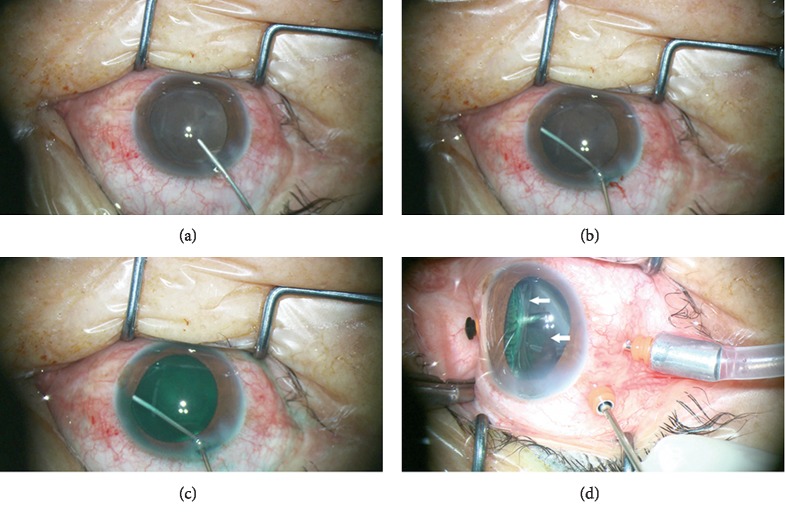
Photographs showing irrigation fluid flow in the posterior chamber irrigation procedure during cataract surgery on a vitrectomized eye. The eye had recurrent retinal detachment after primary vitrectomy and thus required secondary vitrectomy. Fluid flow was visualized using an injection of indocyanine-green solution. The infusion cannula position is shown in Figure 1. (a) A slightly deep anterior chamber was formed with cohesive viscoelastic tamponade. (b-c) Hypotony of vitreous cavity occurred after I/A. Then, the vitreous cavity was infused in the anterior to posterior direction. (b) With low posterior segment pressure, most of the irrigation fluid went posteriorly to the vitreous cavity rather than anteriorly flushing out the viscoelastic. Thus, the anterior chamber did not become shallow during irrigation. (c) As the vitreous pressure increased, reflux of fluid forced the outflow of viscoelastic from the main corneal incision. (d) The ciliary processes, lens capsule, and pars plana (white arrow) were observed to be stained green. The staining provides evidence of irrigation through the zonular fibers to the posterior chamber.

Anterior-to-posterior fluid flow during the irrigation procedure was traced with 2.5 mg/mL indocyanine-green (ICG, 25 mg indocyanine-green in 25% glucose solution 10 mL) irrigation in one cataractous eye, which subsequently required additional vitrectomy and staining of the epiretinal membrane to treat recurrent retinal detachment (Figure 2). In the presence of low vitreous cavity pressure, irrigation flow was traced posteriorly into the posterior chamber and then into the vitreous cavity rather than anteriorly flushing out the viscoelastic. Thus, the anterior chamber did not become shallow during irrigation. The irrigation was terminated once the posterior pressure increased and reflux of fluid forced the outflow of viscoelastic from the main corneal incision.

All the patients were routinely examined one day, one week, one month, and three months after surgery. Main outcomes included intraoperative performance with the irrigation technique, postoperative best-corrected Snellen visual acuity, anatomy, and complications. (Table 1).

## 3. Results

The posterior chamber irrigation technique was performed on ten eyes from nine patients during phacoemulsification cataract surgery. Nine eyes with an attached retina received phacoemulsification and in-the-bag IOL implantation, and two eyes of these eyes received vitrectorhexis to remove posterior capsule plaque after IOL implantation. One eye with recurrent retinal detachment had phacoemulsification followed by a PPV with a silicone oil tamponade.

**Figure 3:**
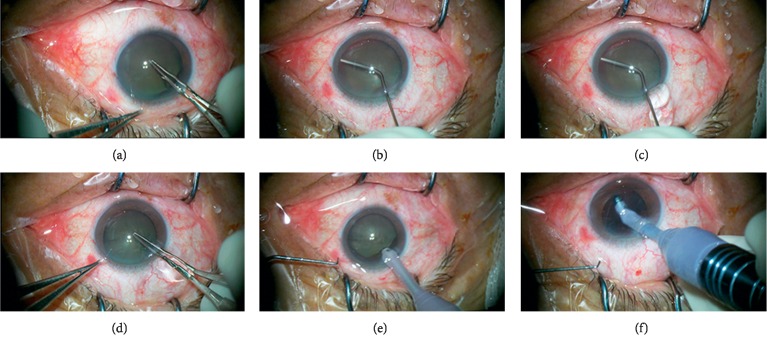
Photographs of the posterior chamber irrigation technique prior to capsulorhexis in postvitrectomy phacoemulsification cataract surgery (patient 7). (a) The vitrectomized eye showed a low vitreous support with posterior retropulsion of iris-lens diaphragm after viscoelastic tamponade in anterior chamber. Capsulorhexis was difficult. (b) The vitreous cavity was irrigated via a 26-gauge flushing cannula injecting balanced salt solution under the iris root. (c) The vitreous cavity pressure surmounted the anterior chamber pressure and viscoelastic flowed out from the main corneal incision. (d) Capsulorhexis was performed smoothly. (e) At the entry of phaco probe, anterior chamber depth remained stable. (f) The anterior chamber was still stabilized throughout the I/A step.

During cataract surgery, the irrigation procedure was performed before capsulorhexis in four eyes (Figure 3), before phacoemulsification in four eyes, after phacoemulsification (before I/A for cortex removal) in two eyes, before IOL implantation in six eyes, and after IOL implantation (before I/A for viscoelastic removal) in three eyes. The procedure was used three times in two eyes, twice in five eyes, and once in three eyes. After the irrigation procedure, posterior segment support was enhanced and the anterior chamber was stabilized throughout the surgery (see [Supplementary-material supplementary-material-1]). None of the patients exhibited signs of persistent posterior segment hypotony, choroidal exudation or hemorrhage, zonular dehiscence, collapse of globe, choroidal exudation or hemorrhage, dropped nucleus fragment, or iris trauma.

Reasonable visual improvement and good anatomic outcomes were observed in all eyes at the three-month follow-up visit. IOLs were centered in the capsular bag, and the retinopathy was stable and intraocular pressure normal.

## 4. Discussion

Anterior segment methods have been established for a safer phacoemulsification cataract surgery after PPV [12, 14, 16], but they could not balance fluid flow and restore vitreous support very well [14], or they were only used for phacoemulsification and I/A steps [16]. The major contribution of the posterior chamber irrigation technique is that it provides viable balance of anterior and posterior segment pressures to enhance vitreous cavity support and stabilize anterior chamber depth throughout cataract surgery after PPV.

The posterior chamber irrigation procedure via an anterior segment approach quickly restores vitreous cavity volume when pressure on the anterior and posterior sides is balanced. The underlying principles are as follows: (1) the amount of viscoelastic tamponade in the anterior chamber sets the volume and pressure for anterior-to-posterior irrigation to the vitreous cavity; (2) the pathway of transzonular fluid flow depends on the pressure disparity between the anterior chamber and the posterior segment. Thus, when the vitreous cavity volume is inadequate, the irrigation distributes more posteriorly than anteriorly. Once the vitreous pressure reaches the anterior chamber pressure, reflux of fluid flushes out the viscoelastic. In our experience, a slightly deep anterior chamber with viscoelastic tamponade may be optimal. Too much viscoelastic may cause a pressurized eye and inadequate vitreous volume to accommodate liquid flow-in. Conversely, a shallow anterior chamber with less viscoelastic allows excessive fluid flow into the vitreous cavity, which may induce hypertension of posterior segment, anterior elevation of iris-lens diaphragm, and prolapse of iris, mimicking the acute fluid misdirection syndrome [18]. Dispersive viscoelastic was not used in our procedures, given its weakness in chamber maintenance. Importantly, the procedure presented herein is simple to be performed during the surgery. It is especially convenient in the following surgical steps: before capsulorhexis, before phaco, before and after IOL insertion, and before I/A for viscoelastic removal, because the anterior chamber has been prefilled with the proper amount of viscoelastic. Special instruments are not needed. The frequency and amount of irrigation should be adjusted according to posterior segment pressure during cataract surgery. Redundant irrigation is to be avoided in eyes that have substantial volume of vitreous after a limited vitrectomy, e.g., epiretinal membrane removal.

The irrigation technique provided intraoperative stability of the anterior chamber with no complications. Without adequate vitreous support, rupture of the posterior capsule and nucleus drop can occur at different times [12]. Thus, sufficient posterior segment support is necessary before all major surgical steps, such as capsulorhexis, phacoemulsification, I/A, and IOL implantation [3, 4, 12, 13]. The irrigation procedure prior to these major surgical steps enhanced posterior support for the posterior capsule and zonular fibers, consequently enabling capsulorhexis on a stabilized anterior capsule plane, facilitating IOL insertion without stretching the zonules, and preventing hypotony and choroidal exudation intra- and postoperatively. Furthermore, preinfusion of the vitreous cavity guarded against a sudden deepening of anterior chamber at hydrodissection, phacoemulsification or I/A, and avoided abrupt posterior capsular rupture or zonular dehiscence. The procedure can be used effectively even for hard cataracts. Repeating the procedure for no more than four times maintained vitreous pressure throughout the phacoemulsification cataract surgery.

Iris irritation and damage to zonular fibers were concerns using our technique; however, such problems did not occur in this case series. The technique is not designed for eyes with extensive zonular deficiency, as fluid rush may lead to further zonular dehiscence or fluid misdirection. To minimize the risk of damage during irrigation, we recommend an adequate viscoelastic tamponade, gentle irrigation dispersion under the iris, and avoidance of injection toward the region with weak zonules.

A limitation of the current study is the application of the procedure to a limited number of cases that needs to be evaluated in future studies on more cases with different clinical scenarios. Real-time intraocular pressure during cataract surgery was not measured using a tonometer which could be beneficial in future studies. Low vitreous support was reflected in real time by a soft scleral wall and excessive bowing of the iris with a deep anterior chamber. In our experience, these symptoms are reliable in judging the timing of using the irrigation procedure during cataract surgery.

In conclusion, the intraoperative posterior chamber irrigation effectively restored vitreous cavity support in cataract surgeries after PPV. The simple maneuver allowed management of fluid flow for a safe phacoemulsification cataract surgery in our small cohort of patients. The safety of our technique in different clinical scenarios still needs to be confirmed in larger studies. Nevertheless, this anterior segment technique is an alternative to the technique of pars plana infusion.

## Figures and Tables

**Table 1:** Characteristics of patients receiving posterior chamber irrigation procedure during phacoemulsification after the previous vitrectomy.

Case	Gender	Age (yrs)	Eye	BCVA (pre-op)	AL (mm)	Nuclei grading	Retinopathy	Previous surgeries	Tamponade	Interval between PPV and phaco (mos)	Time phase of preirrigation	BCVA (3 months post-op)
1	M	49	od	Counting fingers	23.1	3	PDR	(1) PPV	(1) Silicone oil	7	Before phaco; after phaco; after IOL implantation	20/70
								(2) Silicone oil removal	(2) Expanding gas			
			os	Counting fingers	22.9	3	PDR	(1) PPV	(1) Silicone oil	4	Before phaco	20/100
								(2) Silicone oil removal	(2) Expanding gas			
2	M	62	os	Counting fingers	25.2	4	RRD	PPV	Expanding gas	29	Before capsulorhexis; after IOL implantation	20/200
3	M	57	os	20/200	29.4	4	PM, RRD	PPV	Expanding gas	28	Before capsulorhexis; before IOL implantation	20/70
4	F	54	os	20/200	24.5	3	RRD	PPV	Expanding gas	38	Before IOL implantation	20/70
5	M	59	os	20/400	29.4	3	PM, RRD	PPV	Expanding gas	37	Before IOL implantation; after IOL implantation	20/50
6	F	49	od	HM	23.2	2	MH, RRD	PPV	Expanding gas	3	Before capsulorhexis; before IOL implantation	20/200
7	M	62	od	20/200	28.3	4	PM, RRD	PPV	Expanding gas	144	Before capsulorhexis, before IOL implantation	20/50
8	M	39	od	20/200	30.1	3	PM, RRD	(1) SB	(1)No tamponade	48	Before phaco; after phaco; before IOL implantation	20/70
								(2) PPV	(2) Expanding gas			
9	M	49	od	HM	29.6	4	PM, RRD	(1) PPV	(1) Silicone oil	36	Before phaco	20/200
								(2) Removal of silicone oil	(2) Air			

AL, axial length; BCVA, best-corrected visual acuity; F, female; M, male; MH, macular hole; PM, pathologic myopia; IOL, intraocular lens; HM, hand move; RRD, rhegmatogenous retinal detachment; phaco, phacoemulsification; PDR, proliferative diabetic retinopathy; PPV, pars plana vitrectomy; SB, scleral buckle.

## Data Availability

The data are available if required.

## Supplementary Materials

Supplementary Materials Supplementary Video 1: the video shows the intraoperative posterior chamber irrigation technique during phacoemulsification cataract surgery on patient 7 (Table 1) who developed a brunescent cataract after a vitrectomy. The irrigation technique was applied twice in presence of low vitreous cavity support, once during capsulorhexis and another before IOL implantation.
